# Non-Coding RNAs: Functional Aspects and Diagnostic Utility in Oncology

**DOI:** 10.3390/ijms14034934

**Published:** 2013-03-01

**Authors:** Taiho Kim, Armin Reitmair

**Affiliations:** Nesher Technologies, Inc., 2100 W. 3rd St. Los Angeles, CA 90057, USA; E-Mail: tkim@neshertech.com

**Keywords:** ncRNAs, miRNAs, biomarkers, cancers, SMD, ALEX

## Abstract

Noncoding RNAs (ncRNAs) have been found to have roles in a large variety of biological processes. Recent studies indicate that ncRNAs are far more abundant and important than initially imagined, holding great promise for use in diagnostic, prognostic, and therapeutic applications. Within ncRNAs, microRNAs (miRNAs) are the most widely studied and characterized. They have been implicated in initiation and progression of a variety of human malignancies, including major pathologies such as cancers, arthritis, neurodegenerative disorders, and cardiovascular diseases. Their surprising stability in serum and other bodily fluids led to their rapid ascent as a novel class of biomarkers. For example, several properties of stable miRNAs, and perhaps other classes of ncRNAs, make them good candidate biomarkers for early cancer detection and for determining which preneoplastic lesions are likely to progress to cancer. Of particular interest is the identification of biomarker signatures, which may include traditional protein-based biomarkers, to improve risk assessment, detection, and prognosis. Here, we offer a comprehensive review of the ncRNA biomarker literature and discuss state-of-the-art technologies for their detection. Furthermore, we address the challenges present in miRNA detection and quantification, and outline future perspectives for development of next-generation biodetection assays employing multicolor alternating-laser excitation (ALEX) fluorescence spectroscopy.

## 1. Introduction

When the human genome was sequenced in 2001, it was clear that knowing the “code” was only the beginning. The prevailing thought that only five to 10% of the human DNA was actually being used became challenged with the advent of tiling resolution genomic microarrays and whole genome and transcriptome sequencing technologies. Now it was discovered that at least 75% of the genome is actively transcribed [1–3], which dramatically changed the perception of the genome. Evidence suggests that the proverbial “dark matter” of the genome, which reveals extensive antisense, overlapping and non-coding RNA (ncRNA) expression [4–17], may in fact play a major biological role.

The discovery of a vast amount of new RNAs has surely increased our appreciation of the complexity of mammalian genomes and transcriptomes, as well as many other aspects of biology including transcriptional and posttranscriptional regulation of gene expression. The latest statistics available by the time of this review from GENCODE, the reference human genome annotation for The ENCODE Project (Encyclopedia of DNA Elements), show a total number of 55,123 genes, consisting of 20,070 protein-coding, 12,393 long non-coding RNA genes, 9173 small non-coding RNA genes, 13,123 pseudogenes, as well as 364 immunoglobulin/T-cell receptor gene segments (GENCODE freeze Version 13; March 2012, GRCh37 [18]). Without doubt, the catalogue of RNA types has expanded, possibly putting RNA on a par with the functional importance of proteins [19–24].

Depending on the type of ncRNA, transcription can occur by any of the three RNA polymerases (RNA Pol I, II, or III). In general, ncRNAs can be categorized as either short or long. Long ncRNAs (lncRNAs) are processed in full from their templates and range in length from 200 nt to ~100 kb. LncRNAs are mRNA-like transcripts, however, they are poorly conserved and do not function as templates for protein synthesis. Non-coding RNAs shorter than 200 nt are usually considered small ncRNAs, which include small-interfering RNAs (siRNAs), micro-RNAs (miRNAs), small nucleolar RNAs (snoRNAs), PIWI-associated RNAs (piRNAs), repeat-associated short interfering RNAs (rasiRNAs), promoter-associated small RNAs (PASRs), transcription initiation RNAs (tiRNAs), telomere specific small RNAs (tel-sRNAs), tiny RNAs, and cryptic unstable transcripts [22,25,26]. The well-described miRNAs, ranging between 19 and 24 nt in length, serve as important regulators of gene expression and as intricate components of the cellular gene expression network [27–30]. miRNAs modulate gene expression post-transcriptionally by binding to complementary sequences in the coding or untranslated regions (UTRs) of messenger RNAs (mRNAs), and then affect the translation and/or stability of thesemRNAs. Most known functions of miRNAs relate to negative gene regulation.

In various human diseases, the alteration of several ncRNA types has been reported. Of all the ncRNAs, miRNAs are the most widely studied and characterized. The latest miRBase database (miRBase 19, released in August 2012), which provides a searchable online repository for published miRNA sequences and associated annotation, lists 2019 unique mature human miRNAs. Despite the large number of identified miRNAs, however, the scope of their roles in regulating gene expression is not fully understood. Dysregulated expression of microRNAs in various tissues has been associated with a variety of diseases, including cancers (reviewed below), arthritis [31–35], neurodegenerative disorders [36–41], and cardiovascular diseases [42–49], and it has been shown that miRNA signatures can be used as novel biomarkers, potentially offering more sensitive and specific tests than those currently available for early diagnosis of cancer and other diseases.

## 2. Non-Coding RNAs in Oncology

Expression profiling of human tumors based on the expression of miRNAs and other short or long ncRNAs has identified signatures associated with diagnosis, staging, progression, prognosis, and response to treatment [50–53]. An overview of different types of long and short ncRNAs implicated in human cancers is given in Table 1. Long ncRNAs are distinguished by more tissue-specific expression from microRNAs and protein-coding mRNAs, which are often expressed in multiple tissue types. Although the underlying mechanism for lncRNA tissue specificity is unclear, recent studies of chromatin confirmation show tissue-specific patterns, which may affect ncRNA transcription [54,55]. Examples of recently described lncRNAs displaying a clear relationship with cancer include HOX antisense intergenic RNA (HOTAIR), metastasis associated lung adenocarcinoma transcript 1 (MALAT1), prostate cancer gene 3 (PCA3), several other oncogenic and tumor-suppressor lncRNAs, as well as transcribed-ultraconserved regions (UCRs).

HOTAIR was shown to play a role in cancer metastasis and may be an indicator of poor prognosis in patients with primary breast cancer [56]. Elevated expression was observed in both primary and metastatic breast cancer, demonstrating up to 2000-fold increased transcription over normal breast tissue. HOTAIR, located in the mammalian HOXC locus, reprograms chromatin state to promote cancer metastasis by interacting with polycomb repressor complex PRC2, determining PRC2 localization and repression of the HOXD locus [57]. Serving as a scaffold for at least two distinct histone modification complexes, HOTAIR lncRNA binds the PRC2 complex responsible for histone H3K27 methylation and alsolysine specific demethylase 1 (LSD1), a histone lysine demethylase that mediates enzymatic demethylation of H3K4Me2 [58].

MALAT1 was identified using a subtractive hybridization approach to determine differences in gene expression between primary non-small cell lung cancer tumors of patients that were cured by surgery and those that metastasized after surgery [59]. Subsequently, samples from Stage I patients suffering from adenocarcinoma or squamous cell carcinoma were analyzed by qRT-PCR, confirming that expression levels of MALAT1 were significantly higher in metastasizing adenocarcinomas compared to non-metastasizing ones. As no significant differences in gene expression were seen in squamous cell carcinomas, the data provided evidence that the association of lncRNA MALAT1 with metastasis depended on the lung tumor’s histology [59].

PCA3, originally named DD3, was initially identified as overexpressed in prostate tumors relative to benign prostate hyperplasia and normal epithelium using a differential display approach [60]. Further studies later indicated that PCA3 is a very specific prostate cancer gene. Although its mechanism of action is still not known, it proved useful for diagnosis of prostate cancer in urine by adding specificity to the algorithm for prostate cancer diagnosis [61–64]. Development of a PCA3 diagnostic assay for prostate cancer, called the Progensa PCA3 test and marketed by the company Gen-Probe (recently acquired by Hologic, Bedford, MA, USA), represents the most effective clinical translation of a cancer-associated ncRNA, and the rapid timeline of this development—only 10 years between its initial description and a clinical test—suggests that the use of ncRNAs in clinical medicine is only beginning. Nevertheless, successful translation of lncRNA-based biomarkers into clinically useful tests is typically slow, mostly due to the high costs associated with their development.

Other examples of possibly diagnostically useful lncRNAs with oncogenic potential, which upon misregulation could either induce the expression of oncogenes or silence tumor-suppressor genes to prime a cell for transformation, include the H19 gene andantisense ncRNA in the INK4 locus (ANRIL). The H19 gene is exclusively expressed from the maternal allele, has an important role in genomic imprinting during growth and development [65], and is reported to be reactivated during adult tissue regeneration and tumorigenesis. Loss of imprinting at the H19 locus resulted in high H19 expression in various cancers [66–70].

ANRIL, also known as CDKN2B-AS, is located in antisense direction within the p15/CDKN2B-p16/CDKN2A-p14/ARF gene cluster whose tumor-suppressor gene products play central roles in cell cycle inhibition, senescence, and stress-induced apoptosis. Single nucleotide polymorphisms (SNPs), which alter the expression of ANRIL, are associated with many diseases, including various cancers as well as coronary artery disease and diabetes [71]. ANRIL binds to chromobox 7 (CBX7) within the polycomb repressive complex 1 (PRC1) and to suppressor of zeste 12 protein homolog (SUZ12), and through these interactions is involved in transcriptional repression bytargeting of this complex to the chromatin and the establishment of repressive epigenetic marks [72,73]. These findings indicate that lncRNA-mediated silencing of tumor-suppressor genes may be a major mechanism driving tumorigenesis.

In addition to oncogenic lncRNAs, recent studies have identified several examples of tumor-suppressor lncRNAs that could phenotypically affect cells by promoting tumor-suppressor pathways, which, when compromised, prone cells to develop cancer, e.g., a set of low-abundance lncRNAs, produced from the cyclin D1 (CCND1) promoter region, were shown to allosterically modulate the activity of a key transcriptional regulatory sensor of DNA damage signals, a RNA-binding protein known as translocated in liposarcoma (TLS) [74]. The TLS protein changes from an inactive to an active conformation upon binding these lncRNAs. This allows binding and inhibition of enzymatic activities of histone acetyltransferases such as CREB-binding protein (CBP) and p300 [74], thus silencing expression of the cyclin D1 gene which is a cell cycle regulator frequently mutated, amplified, or overexpressed in many tumor types [75].

Furthermore, various lncRNAs have been shown to be induced by the p53 tumor-suppressor pathway in response to DNA damage [8,76]. One of them, long intergenic non-coding (lincRNA)-p21, repressed p53 target genes via association with heterogeneous nuclear ribonucleoprotein K (hnRNP-K). Loss of lincRNA-p21 led to hnRNP-K mislocalization and resulted in a similar tumor-suppressor phenotype than p53 inactivation [76]. Another lncRNA that modulates the effects of a transcription factor is growth arrests-specific 5 (GAS5). It functions by directly interacting with the DNA-binding sites of the glucocorticoid receptor (GR), preventing GR interaction with its cognate glucocorticoid response elements (GREs), and reducing cell metabolismin response to nutrient starvation [77]. It has been shown to be downregulated in breast cancer cells, possibly to keep cancer cells active even under low-nutrient conditions [78,79].

T-UCRs, a new class of lncRNAs, have a tissue-specific expression pattern and are transcribed from ultraconserved regions (UCRs) [80,81]. T-UCRs, which are more conserved than coding genes and are believed to have fundamental functions in vertebrate evolution, were identified when alignments of human, rat, and mouse genomes demonstrated that despite 300 million years of divergent evolution, some genomic regions remained highly conserved (100% identity) [22,82,83]. Often, T-UCRs are found in fragile genomic regions that are usually associated with cancer. In fact, T-UCR expression has been shown to be aberrant in several human carcinomas and leukemias [80]. Deregulated T-UCR signatures are cancer-specific and have prognostic implications. As is also the case for lncRNAs and miRNAs, T-UCRs can act as tumor-suppressor genes or oncogenes, and T-UCR expression is controlled by miRNAs [80].

In respect to short ncRNAs, most studies have focused on miRNAs and expression profiles demonstrated that many miRNAs are deregulated in human cancers [84]. miRNAs differentially expressed between tumors and their normal tissue counterparts have been identified for a variety of tumor types, including prostate cancer [85], breast cancer [86], colorectal carcinoma [87], lung cancer [88], lymphoma [89], papillary thyroid carcinoma [90], glioblastoma [91], hepatocellular carcinoma [92], pancreatic tumors [93], and pituitary adenomas [94]. miRNAs function in carcinogenesis and cancer progression by modulating the expression levels of oncogenes, tumor suppressors and a number of cancer-related genes controlling cell cycle, apoptosis, cell migration, and angiogenesis. For example, the miR-17-miR-92 cluster in T-cell acute lymphoblastic leukemia reduces the level of the transcription factor E2F1 [95,96], the let-7 family represses Ras and Myc oncogenes in cancers [97,98], and the miR-15a/miR-16-1 cluster down-regulates Bcl-2 and induces apoptosis in a leukemic cell line model [99]. The presence of miRNAs in serum and biopsies of patients opened up the exciting prospective of using miRNA signatures as powerful cancer biomarkers, possibly in combination with traditional tumor markers, for diagnosis, staging, progression, prognosis and response to treatment [84,100–106].

## 3. Diagnostic Utility of MicroRNAs as Biomarkers for Cancer Detection and Prognosis

While mRNA expression profiling as well as direct transcriptome sequencing are frequently used for tumor sub-type classification of biopsy materials, microRNA analysis is emerging as a powerful diagnostic weapon for non-invasive early cancer detection and prognosis which will likely be used in the clinic in combination with traditional analytical methods. A number of properties of stable miRNAs, and perhaps other classes of ncRNAs, make them excellent candidate tumor biomarkers, provided that the right combination of markers in a test will result in improved clinical sensitivity and specificity, as well as positive and negative predictive values (PPV/NPV) of a test. As is the case for mRNAs, expression patterns of miRNAs in human cancers appear to be tissue-specific, and miRNA profiles appear to reflect the developmental lineage and differentiation state of tumors. Because a single miRNA can regulate hundreds of downstream genes with different biologic entities, the information gained from miRNA profiling may also provide accurate classification of cancer subtypes. Most importantly, unlike mRNAs that are rapidly degraded in blood, miRNAs are relatively stable, in spite of the fact that high amounts of RNases circulate in blood, as they are protected from endogenous RNase activity, either because they are bound to proteins or packaged into microparticles, which include 50 to 90 nm membrane-bound exosome-like particles, microvesicles, apoptotic bodies and apoptotic microparticles [108–110]. Their release into blood is thought to be related to apoptosis and necrosis of cancer cells in the tumor microenvironment and is also the result of secretion. In addition to serum, miRNAs were also found in other body fluids, including urine, tear, as well as synovial, ascetic, amniotic, and cerebrospinal fluids [61,111–114]. Lastly, miRNAs can be quantitatively measured in human sera or plasma using established techniques such as quantitative reverse transcription polymerase chain reaction (qRT-PCR) or exiting new technologies on the horizon involving multiplex single molecule detection (SMD).

Numerous studies have already identified free circulating miRNAs that are differentially expressed between cancer patients and healthy donors. These miRNAs were found to be either diagnostic or prognostic, and some of them are showing potential roles as predictive factors or drug targets. Important studies on circulating miRNAs in relation to diagnosis and prognosis are reviewed below, and identified miRNAs, or signatures thereof, are summarized according to primary tumor types in Table 2. In this review, we have focused on circulating miRNAs in four major cancers, *i.e.*, prostate, breast, lung, and colorectal cancers, although differentially expressed circulating miRNAs were identified in many more cancer types, including ovarian, esophageal, liver, squamous cell, gastric, hepatocellular, pancreatic, and renal cancers, as well as in hematological malignancies [115–117]. Future, large-scale studies, however, will be needed to determine which markers, or marker combinations, may be suitable for reliable cancer sub-typing.

### 3.1. Prostate Cancer

A comprehensive study examining circulating miRNAs as biomarkers was performed by Mitchell *et al.*[118] including a comparison of source-related differences in miRNA expression. No significant differences between miRNAs in plasma and serum were detected. However, this was limited to only four miRNAs (miR-15b, miR-16, miR19b, miR-24), which might not be representative of all serum-bound miRNAs [118,119]. In contrast to other RNA molecules, mature miRNAs seem to exhibit high stability: Even after incubation at room temperature for up to 24 h, miR-15b, miR-16, and miR-24 were detectable in serum without any apparent degradation [118]. Also under extreme conditions, endogenous miR-15b, miR-16, and miR-24 conserved their high stability, as DNase, RNase, and multiple freeze/thaw cycles did not damage their structure [118,120]. Taking advantage of the observed stability of tumor-derived miRNAs in circulating blood, Mitchell *et al.* evaluated the expression of miR-141 in a case-control cohort of serum samples. They found a remarkably higher expression of miR-141, as detected by qRT-PCR, in prostate cancer than in control samples. Most importantly, at serum levels of >2510 copies per microliter, individuals with cancer were detected with 100% clinical specificity (however, only at 60% clinical sensitivity), allowing to distinguish patients with prostate cancer from healthy controls [118]. Another leading blood-based miRNA tumor marker candidate identified in this study was miR-125b, which promotes cell death by targeting spindle assembly checkpoint gene MAD1 and modulating mitotic progression [121] and was also recently shown to be a marker predicting chemoresistance in breast cancer [122]. miR-141 is a member of the miR-200 family, which functions to repress the epithelial to mesenchymal transition (EMT) by targeting the ZEB family of transcription factors [123]. It was recently shown to act on ovarian tumorigenesis by targeting p38α and modulating the oxidative stress response [124], while circulating plasma miR-141 was also identified as a novel marker for metastatic colon cancer predicting poor prognosis [125].

Brase *et al.*[126] screened miRNAs in serum samples from patients with metastatic and localized prostate cancer. Various miRNAs were highly abundant in the sera of patients with metastatic disease, including miR-141, miR-375, miR-9-3p, miR-200b and miR-516a-3p. Further validation identified circulating miR-141 and miR-375 to be the most pronounced markers for high-risk tumors. Their levels also correlated with high Gleason score or lymph-node positive status. miR-375 was recently shown to directly target the transcriptional coactivator YAP1 in neuroendocrine-lung cancers [127], and was identified as a tumor suppressor gene in human papillomavirus-mediated cervical cancers [128].

In order to analyze the expression pattern of circulating miRNA in serum with localized prostate cancer (PCa), benign prostate hyperplasia (BPH) and healthy individuals, Mahn *et al.*[129] investigated the expression pattern of selected oncogenic miRNAs in serum and demonstrated that a signature comprising miR-26a, miR-32, miR-195, and let-7i was able to discriminate between PCa and BPH patients. Also, they observed that the tissue miRNA levels correlated with preprostatectomy miRNA levels in serum, and that serum miRNA decreased after surgery, indicating a tumor associated release of miRNA [129].

A recent study identified 16 miRNAs, including miR-141, miR-200b, and miR-375, at differential levels in the plasma of men with localized or metastatic prostate cancer [130]. These studies suggest that serum/plasma miRNAs could be used to predict high-risk tumors and, as such, be a useful complement to the current prognostic armamentarium. In particular, the robust association of miR-141, miR-200b, and miR-375 with metastatic disease is noteworthy: These markers could potentially be applied at the time of diagnosis to identify patients with aggressive disease/micrometastases, and/or to predict recurrence following primary treatment.

Combining information on ncRNAs with that on other types of biomarkers may be able to improve cancer risk assessment, detection, and prognosis. Although the use of the traditional protein-based tumor marker prostate specific antigen (PSA) for early detection and staging of prostate cancer as well as monitoring responses to surgical, hormonal and radiation therapy is well established [131–133], one of the major limitations are false-positive results [134–136]. As there is an urgent need to increase the diagnostic specificity of prostate cancer tests, while maintaining—or even increasing—sensitivity, combination of a miRNA signature, including e.g., circulating miR-141, miR-125b, miR-200b, and miR-375 [118,126,130], with traditional protein markers, could translate into a better diagnostic tool with higher specificity, sensitivity, and accuracy, suitable for population-wide screening efforts.

### 3.2. Breast Cancer

Using a qRT-PCR-based approach, levels of a panel of seven candidate miRNAs were quantified in tissue and blood specimens of patients with breast cancer and age-matched control individuals [137]. miR-195 and let-7a were found to be expressed higher in breast cancer patients than in controls, with a mean fold change of 19 and 11, respectively. Additionally, the levels of these two miRNAs decreased significantly after curative tumor resection, and specific miRNAs correlated with certain clinicopathological variables, namely nodal status and estrogen receptor status. These findings suggested that circulating miRNAs have potential as novel breast cancer biomarkers and may prove useful in clinical management during the perioperative period.

Employing a microarray-based approach, Yan *et al.* identified miR-21 overexpression in human breast cancer to be associated with advanced clinical stage, lymph node metastasis and poor prognosis [138]. Subsequently, in a larger study evaluating miR-21 expression in the serum of 102 breast cancer patients and 20 healthy controls, this miRNA was found to be higher expressed in patients, especially in Stage IV breast cancer [139].

Zhu *et al.* found higher miR-155 expression levels in serum samples from women with progesterone receptor positive tumors than tumors that were negative for this nuclear receptor [140], concluding that miR-155 may be differentially expressed in the serum of women with hormone sensitive compared to women with hormone insensitive breast cancer. Another study compared breast cancer associated miRNAs in serum of localized breast cancer patients after primary tumor surgery, metastasized breast cancer patients, and healthy controls [141]. miR-10b, miR-34a and miR-155 could discriminate metastasized breast cancer patients from controls, and the latter was higher expressed in localized breast cancer patients than healthy controls, but also higher than in metastasized breast cancer patients.

Zhao *et al.* conducted a study that reported circulating miRNAs as potential biomarkers of early stage breast cancer with different results for Caucasian American (CA) *versus* African American (AA) women [142]. After comparing levels of circulating miRNAs in plasma samples of 20 patients with early stage breast cancer and 20 matched controls, they reported 17 upregulated and 14 downregulated miRNAs in the 10 CA women and nine upregulated and nine downregulated miRNAs in the 10 AA women. Furthermore, they were able to link these differentially expressed miRNAs to specific pathways using target prediction algorithms.

In a recent study, Schrauder and colleagues performed microarray-based miRNA profiling on whole blood from 48 breast cancer patients at diagnosis along with 57 healthy individuals as controls [143]. All cancers were histologically confirmed as early stage invasive ductal carcinoma of the breast. In their microarray panel of 1100 miRNAs they found that 59 miRNAs were significantly differentially expressed in whole blood from cancer patients compared with healthy controls, and that 13 and 46 miRNAs were significantly up- or down-regulated, respectively. A set of 240 miRNAs that was evaluated by radial basis function kernel support vector machines and 10-fold cross validation yielded a specificity of 78.8%, and a sensitivity of 92.5%, as well as an accuracy of 85.6%, suggesting that miRNA profiling in whole blood has potential as a novel method for early stage breast cancer detection [143].

### 3.3. Lung Cancer

Wei *et al.* showed that plasma levels of miR-21 were significantly higher in non-small cell lung cancer (NSCLC) patients compared to controls, and that miR-21 can serve as a circulating tumor biomarker for the early diagnosis of NSCLC [144]. Foss *et al.* identified miR-1254 and miR-574-5p serum-based biomarkers as potential minimally invasive screening and triage tools for subsequent diagnostic evaluation of early-stage NSCLC [145]. It was possible to discriminate tumor samples from controls with 82% and 77% sensitivity and specificity, respectively, as judged by the use of a receiver operating characteristic (ROC) curve. Another study reported that miR-155, miR-197 and miR-182 in the plasma of lung cancer patients, including Stage I cancers, were significantly elevated compared to controls [146]. The combination of these three miRNAs yielded 81.3% sensitivity and 86.8% specificity in discriminating lung cancer patients from controls.

In a comparison of a pooled serum sample of lung cancer patients with healthy controls, 28 miRNAs were found to be downregulated and 63 miRNAs were upregulated compared to controls [111]. Two of the highest expressed miRNAs, miR-25 and miR-223, were validated by qRT-PCR in an independent set of lung cancer sera and normal sera and also found to be higher expressed in these patients [111]. A different approach was used by Silva *et al.* who preceded their tests by an EpCAM (epithelial cell adhesion molecule)-based immunomagnetic enrichment step [147]. Out of 365 candidates, no miRNAs were found to be upregulated in patients as compared with 20 in the controls, but 10 miRNAs were downregulated. Three of these were differentially expressed in the validation step as well, and lower levels of let-7f were associated with shorter overall survival, while patients with lower levels of miR-30e-3p had shorter disease-free survival, without difference in overall survival [147].

In another study, genome-wide serum miRNA expression analysis was used to investigate the role of serum miRNA in predicting prognosis of NSCLC. Eleven serum miRNAs were found to be altered more than five-fold between longer-survival and shorter-survival groups [148]. Levels of four miRNAs (*i.e.*, miR-486, miR-30d, miR-1 and miR-499) were significantly associated with overall survival, suggesting that this four-miRNA signature may serve as a noninvasive predictor for the overall survival of NSCLC. Bianchi and colleagues reported a 34 circulating miRNA signature, which is able to identify asymptomatic high-risk individuals with early lung cancer and distinguish malignant lesions from benign nodules revealed by low-dose spiral chest computed tomography (LDCT) [149]. Their findings supported and confirmed the results of Boeri *et al.*[150], which reported a 13 circulating miRNA diagnostic signature of NSCLC that could differentiate aggressive from indolent tumors detected by LDCT with approximately 80% accuracy. They further showed that the signature actually appears months before NSCLC can be diagnosed by LDCT in patients whose tumors presented with aggressive clinical behavior. The results reported by the two groups have important clinical implications. They both demonstrated that non-invasive circulating miRNA signatures are able to distinguish between malignant and benign lesions on LDCT, and to differentiate the aggressive subgroup among the entire population of enrolled patients. This represents an important step forward in clinical practice as it may reduce unnecessary surgical intervention and has the potential to serve as a non-invasive screening tool for early lung cancer diagnosis. The miRNA diagnostic signatures, in conjunction with, or even independent from the LDCT screening may represent a new milestone in early lung cancer diagnosis.

### 3.4. Colorectal Cancer

Several studies have looked into the occurrence of selected candidate miRNAs in serum or plasma of colorectal cancer (CRC) patients. Chen *et al.*[111] demonstrated 69 miRNAs in the serum of patients with colorectal cancer that were not detectable in the serum of healthy controls. Moreover, they identified a unique expression profile of 14 serum miRNAs for colorectal cancers that were not present in a different lung cancer group, confirming tissue specificity. Employing qRT-PCR, Ng and colleagues examined the expression levels of miRNAs in plasma from a series of CRC patients and controls [151]. In a training population of 25 colorectal cancer patients and 20 healthy controls, the expression levels miR-92 and miR-17-3p were found to be elevated in the plasma of colorectal cancer patients. They then analyzed a validation cohort of 90 colorectal cancer patients and 50 healthy controls and found that the expression of miR-92 in plasma could distinguish colorectal cancer patients from healthy control patients with 89% sensitivity and 70% specificity. Additionally, both miR-92 and miR-17-3p were not expressed higher in patients with gastric cancer or inflammatory bowel disease, confirming their specificity [151]. Using a microfluidic array technology (Applied Biosystems) Kanaan *et al.* determined levels of 380 miRNAs in CRC patients from whom cancer and adjacent normal tissue were collected [152]. They identified 19 miRNAs that were dysregulated in CRC tissue and evaluated those in an independent plasma test set consisting of 20 CRC patients with 20 age-, and race-matched subjects without CRC. They found that miR-21 was able to differentiate CRC patients from controls with 90% specificity and sensitivity.

A larger study looked at samples from 120 primary colorectal cancer patients and 37 advanced adenoma patients, both taken before surgery, and compared them to 59 age-matched healthy controls which were confirmed to be without colorectal cancer [153]. Two miRNAs, miR-29a and miR-92a, were identified from a training set and confirmed in the larger validation set to be upregulated in CRC plasma compared to controls. Also in patients with adenomas, these miRNAs were expressed higher than in controls, but significantly lower than in true cancer patients. Additionally, these two miRNAs decreased after surgery in another 20 colorectal cancer patients, suggesting that these miRNAs are in fact cancer-specific. Combined ROC analyses using these two miRNAs revealed an elevated area under the curve (AUC) of 0.883 with 83.0% sensitivity and 84.7% specificity in discriminating CRC, and AUC of 0.773 with 73.0% sensitivity and 79.7% specificity in discriminating advanced adenomas. Together, the data indicated that plasma miR-29a and miR-92a have strong potential as novel noninvasive biomarkers for early detection of CRC [153]. Furthermore, serum miR-29a alone was shown by Wang *et al.* to be a promising novel marker for early detection of colorectal liver metastasis [154].

Additional studies evaluated microRNAs as prognostic biomarkers for CRC. Using a qRT-PCR-based approach of miRNA amplification directly from plasma without RNA extraction, Pu and colleagues found that direct amplification of plasma miR-221 can be used as a potential noninvasive molecular marker for diagnosis and prognosis of CRC and is correlated with p53 expression [155]. Kaplan-Meier curve assessment showed that elevated plasma miR-221 levels are a significant prognostic factor for poor overall survival in CRC patients, and immunohistochemistry analysis demonstrated a significant correlation between plasma miR-221 level and p53 expression. Cheng *et al.*[125] found that circulating miR-141 was significantly associated with Stage IV colon cancer in a cohort of 102 plasma samples and that combination of miR-141 and carcinoembryonic antigen (CEA), a widely used marker for CRC, further improved the accuracy of detection. Additionally, their analysis demonstrated that high levels of plasma miR-141 predicted poor survival and that miR-141 was an independent prognostic factor for advanced colon cancer. Thus, plasma miR-141 may represent a novel biomarker that complements CEA in detecting colon cancer with distant metastasis, and high levels of miR-141 in plasma are associated with poor prognosis [125].

## 4. MicroRNA Detection Technologies: State-of-the-Art and Future Perspectives

The discovery of non-coding RNAs is opening up a new understanding of basic disease mechanisms and offers exciting prospects for diagnostics and prognostics. Particularly miRNAs have the potential to revolutionize present clinical disease management, e.g., determining cancer classification, estimating prognosis, predicting therapeutic efficacy, maintaining surveillance following surgery, as well as forecasting recurrence. Given the fact that miRNAs are identified as the first class of RNAs stably present in serum, it would also be of great interest for future studies to both understand the biological functions and find other potential applications of serum miRNAs. However, before novel biomarkers can be routinely used in clinical settings, standardized procedures for sample preparation and proper methods for normalization during analysis are of critical importance. Large scale and independent clinical studies will also be required.

There is currently no gold standard for measuring miRNAs expression [156]. Microarrayand qRT-PCR are two of the most common methods for evaluating known miRNAs [156–160] and it is considered good practice to profile miRNAs by microarray followed by validation with qRT-PCR [161]. There is, however, poor overall correlation between microarray- and qRT-PCR–based miRNA expression quantification. Methods that directly detect miRNAs have low sensitivity because of the extremely short sequences and relatively low copy numbers of miRNAs, requiring more input total RNA. Methods that use amplification can be error prone due to the very short and inflexible template characteristics and similarity in sequences within miRNA families. Amplified samples are also more greatly affected by handling errors [160]. Moreover, relative quantification of miRNA expression by qRT-PCR depends on the small nuclear RNA used as an internal control and there is no standard as to which internal control should be used for the normalization of qRT-PCR data, which could result in erroneous conclusions due to inappropriate normalization [162]. Clarity in describing how standardization controls are chosen would also aid data interpretation. Moreover, due to the small amount of circulating miRNAs and the large amount of proteins, miRNA extraction from serum samples is technically demanding. Thus, miRNA quantification and comparison of sample collectives is difficult, and low abundance miRNAs may be missed entirely. The lack of a platform for accurate absolute miRNA quantification limits the cross-comparison of miRNA expression profiles between different methodologies, such as next-generation sequencing and PCR profiling [163].

### 4.1. Microarray-Based Methods

To date the most widely used techniques to study the expression profile of miRNAs are based on microarray analysis. These approaches are particularly attractive for miRNA profiling since they allow multiplexed detection of miRNAs [164]. Microarray technologies are based on the hybridization between target molecules and their respective complementary probes. Oligonucleotide probes are immobilized on a support platform through a covalent link, and fluorescently labeled miRNAs are hybridized to the array. The specific links between miRNAs and probes generate fluorescent signals that are revealed subsequent to washing steps, and are quantified as discrete spots on the slide. This technique is very attractive because it allows the analysis of a large number of miRNAs and at the same time obtaining a disease-specific miRNA expression profile. The important steps in microarray analysis are the design of probes used for capture of miRNA molecules and the labeling procedure for biological samples. Several modifications in both these steps have been introduced recently that have permitted to improve this technique.

The probe design is influenced by a number of matters related to the nature of miRNAs. Indeed, miRNAs are small molecules that represent only a tiny fraction of total cellular RNA with many of them belonging to the same family, differing only by few nucleotides. These characteristics make it difficult to design multiple probes with a suitable melting temperature (Tm), thus optimizing hybridization conditions without compromising specificity. Moreover, because there are often hundreds to thousands of probes in the same miRNA microarray, Tm normalization is absolutely critical.

Different strategies have been proposed to overcome these problems. Recently, locked nucleic acids (LNA) [165] have been used to increase melting temperature, probe affinity for its target, and mismatch discrimination. This approach provides high sensitivity and specificity. Else, Baskerville *et al.* reported a strategy for Tm normalization by adjusting the length of the probes. In this method, appropriate adaptor sequences are linked to either one or both ends of the miRNA molecules and, based on the adaptor sequence, the probe is either appropriately extended or truncated, depending on whether the original Tm is too low or too high [166].

The procedure used for miRNA labeling is another pivotal step for the success of microarray analysis. Different ways for direct or indirect labeling of miRNAs have been proposed [167]. Indirect methods are based on the labeling of the reverse transcribed miRNA or the RT-PCR product. This increases the labeling stability and sensibility. Direct methods (such as the use of guanine reagents, T4-RNA ligase or chemical labeling) are usually easier to use and help avoid errors introduced by the reactions of reverse transcription and PCR amplification, even though they require a considerable amount of RNA (in the order of micrograms).

To date, various companies (such as Affymetrix, Inc., Santa Clara, CA, USA; Agilent Technologies, Inc., Santa Clara, CA, USA; Applied Biosystems, Inc., Foster City, CA, USA; Exiqon A/S, Vedbaek, Denmark; and Rosetta Genomics, Inc., Rehovot, Israel) provide different microarray platforms for miRNA detection with potential applicability in the clinical arena.

### 4.2. qRT-PCR-Based Methods

Among the several advantages of qRT-PCR, widely used for gene expression quantification [168,169], are the high levels of sensitivity, accuracy, and practical ease that make qRT-PCR a powerful tool for miRNA detection. On the other hand, the limitation of this method for miRNA detection is due to the very short length of mature miRNAs. In fact, the first approach used allowed to detect and quantify precursor molecules rather than mature miRNAs [170].

A stem-loop qRT-PCR-based TaqMan assay was developed by Chen and colleagues from Applied Biosystems and is currently commercialized [171]. This approach shows all the advantages of conventional TaqMan qRT-PCR such as high sensitivity. Moreover, using a stem-loop primer during the reverse transcription reaction, this approach is specific for mature miRNA identification and allows discrimination of closely related miRNAs. This method is better than conventional TaqMan qRT-PCR in terms of reverse transcription efficiency and specificity. To date, stem-loop qRT-PCR is successfully and widely utilized to detect miRNA dysregulation in different cancer types [172–175]. Concomitantly, Raymond *et al.*[176] developed a very sensitive SYBR Green qRT-PCR for the detection of mature miRNAs using LNA-modified primers. Both stem-loop and SYBR Green qRT-PCR methods have the disadvantage of being quite costly, however.

As a new, cost-effective qRT-PCR-based alternative method for mature miRNA detection, Sharbati-Tehrani *et al.*[177] proposed a highly specific and sensitive method (called miR-Q), which neither requires the use of fluorophore probes, nor LNA-modified oligonucleotides. miRNAs are first reverse transcribed and simultaneously elongated using a miRNA-specific oligonucleotide with 5′ overhang and then cDNA molecules are amplified using three DNA-oligonucleotides at different concentrations. The cDNA sequence is first detected and elongated by a specific oligonucleotide with 5′ overhang (*short-miR-x-rev*). Exponential amplification is then performed using two terminal universal primers (*MP-fw* &*MP-rev*) [177]. This approach has been utilized, e.g., to quantify miRNAs in different cancer cell lines [177,178]. A different, but very simple and convenient method is based on Poly(A)-Tailed Universal Reverse Transcription [179,180]. In this approach total RNA is first polyadenylated by poly(A) polymerase and then cDNA is synthesized by using a specific primer containing oligo dTs flanked by an adaptor sequence. Finally, the cDNA is amplified using a miRNA-specific primer and a universal primer.

The above approaches are all low throughput methods. However in more recent years, these approaches have been modified by high-throughput miRNA profiling. For example, Applied Biosystems Inc. provides TaqMan Low Density Array cards that simultaneously quantifiy hundreds of miRNAs by TaqMan qRT-PCR reactions using Megaplex™ stem-loop primer pools for the miRNA reverse transcription step. Furthermore, Signosis Inc. has developed a highly sensitive and specific platform that combines oligo-ligation and SYBR green-based qRT-PCR for multiplex miRNA detection. These platforms are particularly attractive since they can be used in clinical settings.

An example to simultaneously quantify different miRNAs in the same qRT-PCR reaction is the use of innovative probes named molecular beacons. Molecular beacons are single-stranded probes with a stem-loop structure that recognize a specific target molecule [181,182]. The complementary sequence to the target is contained within the loop of the molecule, while the stem is formed via annealing of two complementary sequences added to the 3′ and 5′ ends, with a fluorophore linked to the end of one arm and a quencher linked to the end of the other one. Molecular beacons emit fluorescence only when they hybridize with the target, undergoing a spontaneous conformational reorganization that forces the fluorophore and the quencher to move away from each other. This approach is very sensitive to mismatches and, since probes can be linked to different fluorophores, is also helpful to simultaneously detect different target miRNAs. Molecular beacons have also been modified [183] to specifically quantify mature miRNAs. In this case, when the probes hybridize to pre-miRNA or pri-miRNA, its fluorescence is quenched by a guanine in the target sequence, while hybridization of the probe with mature miRNA, which has no complementary guanine, results in fluorescent signal emission. This approach has been recently utilized for detection of miRNAs overexpressed during myogenic differentiation [184].

### 4.3. Deep Sequencing-Based Methods

Inrecent years, innovative deep sequencing technologies, originally used for genomic sequencing [185], have also been applied for parallel sequencing of up to millions of miRNA molecules. These include the 454 Genome Sequencer (Roche Applied Science, Basel CH) based on pyrosequencing, the Illumina Genome Analyzer (Illumina Inc., San Diego, CA, USA) based on the Solexa technology, and the SOLiD platform (Applied Biosystems, Inc., Foster City, CA, USA), a ligation-based sequencing method. For these high-throughput systems, a small RNA cDNA library preparation is critical and involves the following basic steps: (1) total RNA isolation; (2) enrichment for small RNAs; (3) 3′ and 5′ adaptor ligation (platform-specific); (4) reverse transcription; (5) PCR amplification by minimal rounds to avoid library bias; (6) sequencing [159].

For the 454 and in the SOLiD technologies, an adaptor-flanked library is amplified by an emulsion multi-template PCR using a single primer pair, corresponding to the adaptor sequences. One PCR primer is 5′-linked to the surface of micron-scale beads, included in the reaction. After PCR amplification, each bead will bear on its surface PCR products corresponding to a single molecule from the template library. These clonally amplified beads can then be used as templates for the 454 or SOLiD sequencing platforms. For the 454 platform, beads are randomly deposited on the wells of a microarray and sequenced by pyrosequencing. In this approach, during each cycle a single nucleotide is introduced and then a substrate (luciferin, adenosine 5′-phosphosulphate) is added to produce a light signal at wells where the polymerase drives the incorporation of the nucleotide.

Considering the SOLiD platform, beads are used to create a disordered, dense array of sequences, and each sequencing cycle introduces a partially degenerate population of fluorescently labeled octamers. In this population the label correlates with the identity of the central 2 bp of the octamer. Several such cycles will iteratively interrogate an evenly spaced, discontiguous set of bases.

Finally, considering the Solexa technology, an adaptor-flanked library is amplified by a bridge PCR, in which primers are linked to the surface of a solid substrate by a flexible linker. At the end of the PCR reaction different clonal clusters are generated, each containing ~1000 copies of a single member of the starting library. These clusters are then sequenced. Each sequencing cycle includes the simultaneous addition of a mixture of four fluorescently labeled deoxynucleotides modified with a reversibly terminating moiety at the 3′ position. A modified DNA polymerase drives synchronous extension of primed sequences and results are acquired by imaging in four channels.

These strategies allow a fast evaluation of absolute miRNA levels and are also able to identify novel miRNAs, but to date they are still costly. In addition, since errors can be introduced at several steps, limiting the accuracy of the analysis, sequencing results must be validated by alternative methods such as qRT-PCR. In brief, these methods determine the nucleotide sequence by taking a picture every time a new nucleotide is added to the growing strand, thus emitting light [186]. To ensure sufficient light signal intensity for accurate detection of each added nucleotide, these methods typically amplify the fragments through emulsion PCR, or library generation followed by PCR-based cluster amplification. However, amplification can result in sequence errors and some sequences may be preferentially amplified, limiting the ability to accurately quantify relative abundance. These methods can also be less accurate in areas of homopolar (identical) bases.

New techniques to read the sequence derived from a single molecule are currently under development. Limitations of next-generation sequencing include bioinformatic challenges due to large quantities of data, and the high cost of instruments and reagents, although each sample can be bar-coded to allow samples to be mixed and run simultaneously to reduce cost. The third generation of sequencing technologies, currently under development, could eventually provide lower cost options [186,187].

### 4.4. Single Molecule Detection Methods

To maximize clinical information content obtainable from a single small patient sample, SMD technologies may be very promising for development of next-generation medical diagnostics. Although several different SMD methods such as single-molecule real-time sequencing [188], nanopore single molecule counter [189], and single molecule miRNA detection [190] are being evaluated for their potential use in medical diagnostics, here we focus on multicolor alternating-laser excitation fluorescence spectroscopy, a SMD technology which is being advanced at our laboratory and holds promise for absolute biomarker quantification in multiplexed fashion directly in minute patient samples.

Alternating laser excitation fluorescence aided single molecule sorting (hereafter ALEX) was developed as a new and improved, solution-based robust tool for ultrasensitive, -specific, and highly multiplexed quantification of target molecules in complex samples [191–201]. In its original implementation, 2-color (2c) ALEX [191–193], two high-affinity recognition molecules (e.g., DNA oligos, antibodies), which are labeled with different color fluorescence dyes, bind to two mutually exclusive areas on the target molecule of interest. By counting the number of two different color coincident fluorescent bursts in a femtoliter confocal detection volume, the actual number (or concentration) of the target can be derived in a real time fashion. The dual-color coincidence detection by laser alternation allows virtual exclusion of the majority of background noises, eliminating the need for washing steps (necessary e.g., in microarray-based analysis), which, together with elimination of amplification steps, enables substantially improved accuracy of quantification with minimal sample requirements.

We recently successfully expanded 3c-ALEX [201], where molecules are sorted in three-dimensional *S* (Stoichiometry ratios) and *E* (FRET, Förster resonance energy transfer) histograms, further to four-colors (4c-ALEX) [202]. Molecule sorting in multi-dimensional space substantially extends ALEX single-well multiplexing capabilities and permits differentiation of numerous targets simultaneously, especially when exploiting FRET to monitor distances between fluorescent donors and acceptors incorporated at specific sites on individual detector molecules (e.g., DNA oligos) which allows implementation of fluorophore “barcoding”. Furthermore, recent work shows successful combination of microfluidics-based sample handling of nanoliter volumes with ALEX spectroscopy (“single molecule optofluidics” [203]). ALEX-based SMD is thus ideal to minimize removal of precious samples (e.g., synovial fluid, cerebrospinal fluid, blood) for analysis. By directly counting the number of fluorescent bursts, absolute target concentrations can be determined accurately, which is critical for improving standardization of biomarker measurements.

**Figure 1:**
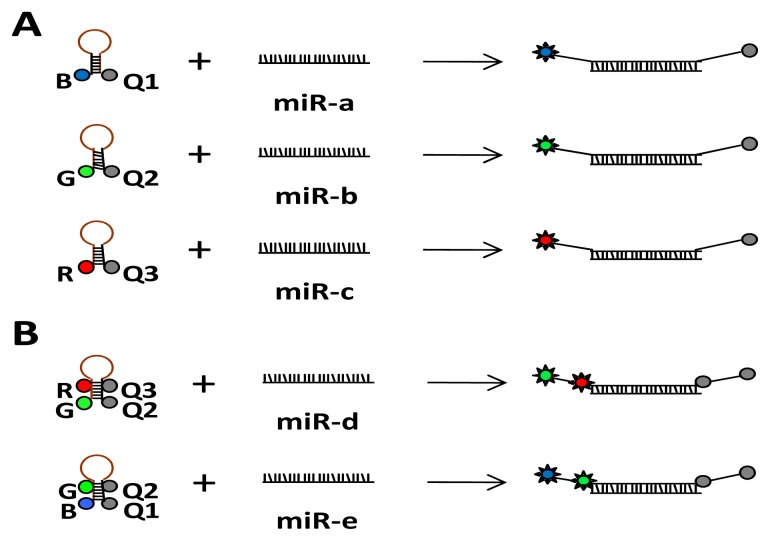
Illustration of a possible molecular beacon probe labeling scheme for multiplexed microRNAs (miRNA) detection (exemplified for three color alternating laser excitation fluorescence aided single molecule sorting (3c-ALEX)). Different signals are produced upon dequenching of individual dye-quencher pairs (**A**) or multiple fluorophore-quencher pairs positioned at distinct Förster resonance energy transfer (FRET) distances between donor and acceptor (**B**).

Multiplexed detection and simultaneous quantification of multiple miRNAs is one of the biggest advantages of using the ALEX detection scheme: The ability to detect multiple species in the same reaction mixture by incorporating multiple fluorescent dye probes with different excitation/emission characteristics in conjunction with multicolor excitation/detection to measure multiple distances between distinct fluorescence probes via FRET and stoichiometry ratios. Without FRET involvement between a donor fluorophore and an acceptor fluorophore, two color (2c) ALEX allows differentiation of two, three color (3c) ALEX differentiation of three, and four color (4c) ALEX differentiation of four miRNA species. Thus, in its simplest implementation for multiplexed miRNA detection and quantification, different emission wavelength signals are produced upon dequenching of individual dye-quencher pairs comprised of different wavelength fluorophore-quencher pairs. To increase multiplexing power, distinct FRET values, specifically designed for each miRNA target, can be generated by placing multiple fluorophore-quencher pairs at distinct FRET distances on each probe (Figure 1). In general, multi-distance analysis towards more complex levels of n-color-ALEX will enable observation of n-component interactions up to [n(n − 1)/2] donor-acceptor pairs and at least three (low, medium, and high FRET) multi-distances per FRET pair within a single biomolecule complex/target-sequence area, allowing full implementation of barcoding for highly multiplexed target detection in a single well.

We recently achieved amplification-free detection of prostate cancer-specific (miR-141 and miR-375) and lung cancer-specific (miR-30d-5p and miR-25-3p) miRNAs at subpicomolar concentrations (T.K. and A.R., unpublished). This may afford ALEX a unique opportunity to improve existing cancer tests (e.g., the popular PSA test, which lacks clinical specificity and leads to overdiagnosis and unnecessary biopsies [204]) by combining protein-based and microRNA-based biomarkers. Furthermore, the technology may be useful for the development of tests for personalized-medicine applications by simultaneously querying multiple single-nucleotide polymorphisms and copy number variants, in addition to the development of biomarker panels for health status assessments with fingerprick-size blood or other patient samples.

The current technology, however, lacks fast-performance, high-throughput, and automated liquid-handling functionalities. To develop a “sample in, answer out” type of analytical instrument that can be used reliably without highly trained personnel will require integration of additional technology components. The feasibility of integrating microfluidics-based sample handling [203], up-front target enrichment procedures [205], and emerging multifoci photon-counting detectors for increased throughput [206,207], offers the exciting opportunity to develop a rapid, fully automated, ultrasensitive, and ultraspecific highly multiplexed clinical diagnostic platform based on ALEX single-molecule spectroscopy. A comparison of the ALEX technology (in its current, research-grade state as well as with future potential after integration of microfluidics and multifoci photon-counting detectors) *versus* different currently available platform technologies for microRNA detection and profiling is shown in Table 3.

## 5. Conclusions

In summary, all of the current and new technologies have benefits and limitations to consider when designing miRNA studies and assays for diagnostic, prognostic, and predictive applications. Results can vary across platforms, requiring careful and critical evaluation when interpreting findings. When costs come down, as they have for genotyping, next-generation sequencing may allow fast and possibly more accurate miRNA profiling. But since patient samples are limited for detection of biomarkers, which are typically present in very low concentrations making serial analysis impractical, the ALEX technology may be well-suited for next-generation diagnostics such as multiplexed evaluation of miRNA expression, along with traditional protein-based markers, in various diseases including cancers. Furthermore, future blood-based tests using only finger-prick size blood samples may offer the exciting possibility to limit the need for venipuncture.

The identification of miRNAs in body fluids, including blood, has triggered substantial excitement in the biomarker field. As discussed, deregulated expression of miRNAs has been extensively described in a variety of diseases, particularly in cancer. Based on their stability and relative ease of detection, circulating miRNAs may become one of the most important parts in the personalized medicine arsenal. So far, many miRNAs have been shown to regulate pathways in cancer and other diseases as part of a genetic and epigenetic network, and have been used to develop diagnostic, prognostic and therapeutic strategies. However, it is becoming clear that a comprehensive understanding of human biology must include both small and large non-coding RNAs. Thus, we are just starting to envision the use of other types of ncRNAs with the same purposes.

## Figures and Tables

**Table 1:** Different types of non-coding RNAs involved in human cancers (modified after Sana *et al.*[107]).

Type	Class	Symbol	Characteristic	Cancer/biological function associations
**Long non-coding RNAs (lncRNAs, ≥200 nt)**	**Long intergenic non-coding RNAs**	lincRNAs	ranging from several hundreds to tens of thousands nts; lie within the genomic intervals between two genes; transcriptional	involved in tumorigenesis and cancer metastasis/involved in diverse biological processes such as dosage compensation and/or imprinting
	**Long intronic non-coding RNAs**		cis-regulation of neighbouring genes lie within introns; evolutionary conserved; tissue-specific expression patterns	aberrantly expressed in human cancers/possible link with posttranscriptional gene silencing
	**Telomere-associated ncRNAs**	TERRAs	100 bp >9 kb; conserved among eukaryotes; synthesized from C-rich strand; polyadenylated; form intermolecular G-quadruplex structure with single-stranded telomeric DNA	possible impact on telomere-associated diseases including many cancers/negative regulation of telomere length and activity through inhibition of telomerase
	**Long non-coding RNAs with dual functions**		both protein-coding and functionally regulatory RNA capacity	deregulation has been described in breast and ovarian tumors/modulate gene expression through diverse mechanisms
	**Pseudogene RNAs**		gene copies that have lost the ability to code for a protein; potential to regulate their protein-coding cousin; created via retrotrans-positions; tissue-specific	often deregulated during tumorigenesis and cancer progression/regulation of tumor suppressors and oncogenes by acting as microRNA decoys
	**Transcribed-ultraconserved regions**	T-UCRs	longer than 200 bp; absolutely conserved between orthologous regions of human, rat, and mouse; located in both intra- and intergenic regions	expression is often altered in some cancers; possible involvement in tumorigenesis; antisense inhibitors for protein-coding genes or other ncRNAs
	**Antisense RNAs**	aRNAs	complementary antisense transcripts	antisense transcripts appear to be a pervasive feature of human cells, which suggests that they are a fundamental component of gene regulation
	**Long stress-induced non-coding transcripts**	LSINCTs	longer than 300 nucleotides; expression is increased in response to the DNA damage-inducing tobacco carcinogen 4-(methylnitrosamino)-1-(3- pyridyl)-1-butanone (NNK)	increased expression in a number of cancer-derived cell lines

**Small non-coding RNAs (<200 nt)**	**MicroRNAs**	miRNAs	18–25 nt; account 1%–2% of the human genome; control the 50% of protein-coding genes; guide suppression of translation; Drosha- and Dicer-dependent small ncRNAs	initiation of various disorders including many, if not all, cancers/regulation of proliferation, differentiation, and apoptosis; involved in human development
	**Small nucleolar RNAs**	snoRNAs	60–300 nt; enriched in the nucleolus; excised from pre-mRNA introns in vertebrates; bind snoRNP proteins	association with development of some cancers/important function in the maturation of other non-coding RNAs, above all, rRNAs and snRNAs; miRNA-like snoRNAs regulate mRNAs
	**Pyknons**		subset of patterns of variable length; form mosaics in untranslated and protein-coding regions; more frequently in 3′ UTRs	expected association with cancer biology/possible link with posttranscriptional silencing of genes, mainly involved in cell communication, regulation of transcription, signaling, transport, *etc*.

**Table 2:** Circulating microRNAs with diagnostic and prognostic utilities in various cancer types (modified after Mostert *et al.*[116]).

Sample type	Method	Patients (*n*)	HDs (*n*)	Normalization procedure based on	Candidate miRNAs (*n*)	Differentially expressed miRNAs	Prognostic	Reference
*Prostate cancer*								

Serum	qRT-PCR	25	25	Spiked-in miRNA	6	*miR-141*	No	Mitchell *et al.*[118]
Serum	qRT-PCR array †	21	None	Spiked-in miRNA	667	69 *miRs* up ††, 0 *miRs* down ††		Brase *et al.*[126]
	qRT-PCR	45	None	Spiked-in miRNA	5	*miR-9*†, *miR-516a-3p*	No	
		116	None	Spiked-in miRNA		*miR-141*, *miR-375*	Yes	
						*miR-200b*	No	
Serum	qRT-PCR	35	20		4	*miR-26a*, *miR-32*, *miR-195*, *let-7i* (only in benign prostate hyperplasia)		Mahn *et al.*[129]
Plasma	qRT-PCR	78	28		742	*16 miRs* including *miR-141*, *miR-200b*, *miR-375*		Bryant *et al.*[130]

*Breast cancer*								

Whole blood	qRT-PCR	148	44	*miR-16*	7	*miR-195* *let-7a*	No	Heneghan *et al.*[137]
Serum	qRT-PCR	102	20	*miR-16*	1	*miR-21*	No	Asaga *et al.*[139]
Serum	qRT-PCR	13	8	*cel-miR-39, has-miR-145*		*miR-155*		Zhu *et al.*[140]
Serum	qRT-PCR	89	29	*miR-16*	4	*miR-10b*, *miR-155* *miR-34a*	No	Roth *et al.*[141]
Plasma	Illumina microarray †	10CA	10 CA	Quantile normalization algorithm	1145	17 *miRs* up, 14 *miRs* down	No	Zhao *et al.*[142]
		10AA	10 AA			9 *miRs* up, 9 *miRs* down		
Whole blood	microarray	48	57		1100	13 *miRs* up, 46*miRs* down		Schrauder *et al.*[143]

*NSCLC*								

Plasma	qRT-PCR	63	30			*miR-21 up-regulation*		Wei *et al.*[144]
Serum	qRT-PCR	11 (profiling) 31 (validation)	11 (profiling) 22 (validation)			*miR-1254, miR-574-5p up-regulation*		Foss *et al.*[145]
Plasma	qRT-PCR	74	68			*miR-155*, *miR-197*, *miR-182*		Zheng *et al.*[146]
Serum	Solexa sequencing †	11 ‡	21 ‡	Total RNA	190	63 *miRs* up, 28 *miRs* down		Chen *et al.*[111]
	qRT-PCR	152	75	Average of HDs	3	*let-7a*, *miR-223* *miR-25*	No	
Exosomes	qRT-PCR array after EpCAM-based enrichment step †	28	20	*miR-142-3p; miR-30b*	365	0 *miRs* up, 10 *miRs* down		Silva *et al.*[147]
	qRT-PCR			*miR-142-3p; miR-30b*	5	*let-7f*, *miR-30e-3p*	Yes	
						*miR-20b*	No	
Serum	Solexa sequencing †	2 × 30 ‡	None	Spiked-in miRNA	101/109 §	3 *miRs* up §, 8 *miRs* down §		Hu *et al.*[148]
	qRT-PCR	303	1	One HD	11	*miR-486*, *miR-1*# *miR-30d*, *miR-499*#	Yes	
Serum	qRT-PCR array	59	69			34 *miRs*		Bianchi *et al.*[149]
Tissues and Plasma	Microarray followed by qRT-PCR	19 (training set), 22 (validation set)				13 *miRs*		Boeri *et al.*[150]

*Colorectal cancer*								

Serum	Solexa sequencing †	11 ‡	21 ‡	Total RNA	190	69*miRs* when compared to healthy controls		Chen *et al.*[111]
	qRT-PCR	152	75	Average of HDs	3	*14 miRs* when compared to a lung cancer group		
Plasma	qRT-PCR array †	25	20	U6	95	*miR-17-3p*, *miR-135b* *miR-92, miR-222 miR-95*		Ng *et al.*[151]
	qRT-PCR	90	50	U6	5	*miR-17-3p* *miR-92*	No	
Tissues and Plasma	Microfluidic array	20	20		380	90% of 19 *miRs* dysregulated in colorectal cancer patient plasma, especially *miR-21*		Kanaan *et al.*[152]
Plasma	qRT-PCR	100	59	*miR-16*	12	*miR-29a* *miR-92a*	No	Huang *et al.*[153]
Serum	qRT-PCR	74 (40 for validation)			3	*miR-29a*		Wang *et al.*[154]
Plasma	qRT-PCR	103	37	Standard curve	3	*miR-221*	Yes	Pu *et al.*[155]
Plasma	qRT-PCR	102				*miR-141*		Cheng *et al.*[125]

† Discovery phase.

‡ Pooled samples.

§ Differentially expressed between long and short survival groups. Higher expressed in short survival group.

# Higher expressed in long survival group.

†† Higher expressed in metastatic compared with localized prostate cancer patients. AA: African–American; CA: Caucasian–American; HD: Healthy donor; miR: miRNA; NA: Not applicable; NSCLC: Non-small-cell lung cancer; qRT-PCR: Quantitative reverse-transcriptase PCR.

**Table 3:** Comparison of platform technologies for microRNA profiling (expanded upon Baker *et al.*[208]).

Characteristic	qPCR *	Microarray *	Sequencing *	4c-ALEX (96 wells; current prototype)	Multifoci (64) 4c-ALEX (384 well optofluidics chip; in development)
**Throughput time**	~6 h	~2 days	1–2 weeks	~2.5 days (each well takes 30 min acquisition time) to ~1 weeks (triplicates)	~3 h (64 times faster data acquisition) to 9 h (triplicates)
**Total RNA required**	500 ng	100–1000 ng	500–5000 ng	~50 ng	~50 ng
**Estimated cost per sample**, **including reagents and supplies**	$400 (754 human microRNAs queried per sample)	$250–$350 (at least 950 microRNAs queried per sample)	$1000–$1300 (theoretically, all microRNAs queried per sample)	$10 (576 microRNAs queried per sample)	$10 (theoretically, all microRNAs queried per sample)
**Dynamic range detected**	Six orders of magnitude	Four orders of magnitude	Five or more orders of magnitude	Four orders of magnitude	Four orders of magnitude
**Ease-of-use**	Easy	Moderate	Difficult	Easy	Easy
**Infrastructure and technical requirements**	Few	Moderate	Substantial	Few	Moderate

Note:

* for qPCR, Microarray, and Sequencing, results were reported by the Association of Biomolecular Resource Facilities. Newer protocols and equipment may have different prices, throughput, output and requirements.
